# Prioritizing Risk in Preparation for a Demonstration Project: A Mixed Methods Feasibility Study of Oral Pre-Exposure Prophylaxis (PREP) among Female Sex Workers in South India

**DOI:** 10.1371/journal.pone.0166889

**Published:** 2016-11-23

**Authors:** Sushena Reza-Paul, Lisa Lazarus, Monika Doshi, Syed Hafeez Ur Rahman, Manjula Ramaiah, Raviprakash Maiya, Venugopal MS, K. T. Venukumar, Sundar Sundararaman, Marissa Becker, Stephen Moses, Robert Lorway

**Affiliations:** 1 Centre for Global Public Health, Department of Community Health Sciences, University of Manitoba, Winnipeg, Canada; 2 Ashodaya Samithi, Mysore, India; 3 Independent Consultant, Chennai, India; University of Toronto, CANADA

## Abstract

**Background:**

HIV prevalence among female sex workers (FSWs) in India remains well above the national average. Pre-exposure prophylaxis (PrEP), a new HIV prevention technology, may help to reduce HIV incidence, but there is a dearth of research that can inform the potential scale-up of PrEP in India. In partnership with *Ashodaya Samithi*, a local sex worker collective, we conducted a feasibility study to assess acceptance of a planned PrEP demonstration project, willingness to use PrEP, and recommendations for project roll-out among FSWs in southern Karnataka.

**Methods:**

From January–April 2015, 6 focus group discussions, 47 in-depth interviews, and 427 interviewer-administered questionnaires were completed by female sex workers. All participants were 18 years of age or older and practiced sex work. Qualitative data were coded for key themes and emergent categories. Univariate descriptive analysis was employed to summarise the quantitative data.

**Results:**

**Qualitative**. PrEP was described as an exciting new prevention technology that places control in the hands of FSWs and provides a “double safety” in combination with condom use. Participants expressed agreement that women who may experience more HIV risk in their occupational environments should be prioritized for enrollment into a demonstration project. **Quantitative**. 406 participants (95%) expressed interest in PrEP. Participants prioritized the inclusion of FSWs under the age of 25 (79%), those who do not use condoms when clients offer more money (58%), who do not consistently use condoms with regular partners (57%), who drink alcohol regularly (49%), and who do not use condoms consistently with clients (48%).

**Discussion:**

This feasibility study indicated strong interest in PrEP and a desire to move forward with the demonstration project. Participants expressed their responses in terms of public health discourses surrounding risk, pointing to the importance of situating PrEP scale up within the trusted spaces of community-based organizations as a means of supporting PrEP uptake and adherence.

## Background

An estimated 2.1 million people are living with HIV in India, with an HIV prevalence of 0.3% in the general adult population [1]. Although HIV prevalence has been declining within the general population over the past decade, key populations such as female sex workers (FSWs) continue to face an HIV burden well above the national average, with a recorded average HIV prevalence of 2.2% [2]. In response, targeted structural interventions which address legal, institutional, social, cultural and economic determinants of HIV risk have led to significant declines in HIV prevalence [3–6]. Despite these noted successes, HIV incidence among FSWs remains a concern. Though incidence data among FSWs in India is limited, in the year 2013, India had an estimated 130,000 new HIV infections and accounted for 38% of new HIV infections in Asia [1]. The challenge lies in further reducing HIV incidence among FSWs. One possible approach may be oral pre-exposure prophylaxis (PrEP), a new HIV prevention strategy that involves the use of antiretroviral drugs to reduce the risk of HIV infection [7]. People who do not have HIV take a daily pill (most commonly tenofovir alone or in combination with emtricitabine) to prevent HIV acquisition from occurring. As of September 2015, the World Health Organization (WHO) recommends that people at “substantial risk” of HIV infection be offered PrEP as an additional prevention choice, as part of comprehensive HIV prevention strategies [8].

Much is known about the clinical efficacy of PrEP, with trials beginning in 2005. Studies have focused on PrEP use among men and transgender women who have sex with men, HIV serodiscordant couples, people who inject drugs, heterosexual men and women, and women at higher risk of HIV exposure [9–19]. Recent findings have found PrEP to be 86% effective among men who have sex with men (MSM) and 96% effective among serodiscordant couples where the HIV-positive partner is on antiretroviral treatment (ARVs) [20–22]. Despite PrEP’s demonstrated efficacy as a new prevention technology, guidance and practices around its implementation and scale up in India remain unclear. For this reason, we have planned a demonstration project in two sites in India: the *Durbar Mahila Samanwaya Committee* (DMSC) at Sonagachi in Kolkata [23–25] and *Ashodaya Samithi* (*Ashodaya*) in Mysore [4, 26–30] to test its “real world” effectiveness if rolled out as part of an actual program. It is planned that PrEP will be provided free of cost throughout the duration of the 16-month demonstration project, and participants will be expected to undergo quarterly health screenings, including pregnancy, HIV and STI testing, as well as participate in behavioural surveys. The outcome of the demonstration project will be used to develop guidelines for the implementation and scale up of PrEP among female sex workers in India.

Prior to moving forward with a demonstration project, it was necessary to consult with the communities who will be asked to participate in the study. Some early PrEP trials with FSWs in Cameroon, Nigeria, Malawi, and Cambodia were cancelled before they started. In Cambodia, concerns were expressed by FSWs about the lack of community involvement, drug safety, and confusion over the right to treatment, especially in instances of HIV infection during study participation [5,31]. In other settings, studies were cancelled due to allegations of inappropriate research standards [5,31]. Feasibility studies can help determine whether an intervention is appropriate for further testing, and can help inform the acceptability, demand, practicability, and implementation of the proposed project [32]. The University of Manitoba’s Centre for Global Public Health, in partnership with *Ashodaya Samithi*, a sex worker collective, conducted a feasibility study to assess acceptance of a PrEP demonstration project, willingness to use PrEP, and recommendations for project roll-out among female sex workers in Mysore and Mandya, Karnataka, India. A similar study was also undertaken by DMSC at Kolkata. In this paper, we present the data from the feasibility study conducted by Ashodaya Samithi.

## Methods

The feasibility study took place between January and April 2015 and followed a sequential mixed methods approach [33,34], drawing on both qualitative and quantitative research methodologies. Participants for the feasibility study included female sex workers (FSWs) who were 18 years of age or older and currently practiced sex work in Mysore and Mandya.

### Study Site

*Ashodaya Samithi* was the first site to be supported through *Avahan*, the India AIDS Initiative of the Bill & Melinda Gates Foundation, in 2004. Its work has been previously published elsewhere [4, 26–30]. Briefly, *Ashodaya* currently has a membership of over 8000 female, male, and *hijra*/transgender sex workers across six districts of the state of Karnataka. *Ashodaya*’s core areas of work include program implementation, outreach and advocacy, sexual and reproductive health-related clinical services, addressing sexual and gender-based violence, capacity building, and participatory research. *Ashodaya* has implemented numerous health programs, such as condom promotion, screening and treatment for STIs, and reproductive health services. Members of the community have also received training to conduct qualitative, quantitative, and mixed methods study designs. Ashodaya has been designated as both a national and global learning site, resulting from the work that they have done in building the capacity of other sex worker organizations. Community engagement, hence, forms a crucial backdrop for interpreting the findings generated through research implemented by this site.

### Awareness Campaigns

As a new HIV prevention strategy, PrEP has yet to be implemented in India and knowledge around it remains quite limited. The initial community consultations revealed very low or no knowledge around PrEP. Therefore, in order to ensure informed decision making by the community around feasibility of PrEP implementation and scale-up, raising awareness around PrEP became critical. A PrEP fact sheet based on the Centers for Disease Control and Prevention (CDC), AIDS Vaccine Advocacy Coalition (AVAC), and Global Network of Sex Work Projects (NSWP) guidelines was prepared and simplified in a language that could be easily understood by the community. Information on PrEP was disseminated through community meetings and one-on-one interactions. Approximately 30 PrEP-related group discussions took place in Mysore and Mandya; each discussion had 25–30 participants. Awareness campaigns lasted for almost nine months. Following this, a feasibility study was undertaken.

### Focus Group Discussions

Focus group discussions (FGD) were conducted in small groups of 6–8 FSWs. A team of trained community and non-community researchers conducted the FGDs in the local language, Kannada. The groups were kept as homogenous as possible using criteria such as socio-economic status, education level, or social networks. Purposive sampling was used by the research team to identify participants by using peer educators’ contact lists. Informed consent was obtained from each participant before the start of the focus group. Discussions focused on exploring (a) knowledge, interests, and concerns around PrEP; (b) the acceptability and willingness for PrEP uptake, including perceptions of HIV risk, as well as barriers and facilitators to uptake and adherence; and (c) inclusion/exclusion criteria for participant enrolment into the demonstration project.

### In-Depth Interviews

Semi-structured in-depth interviews were conducted among FSWs by trained community and non-community researchers. The interviews were conducted in the local language, Kannada. Participants were identified using purposive sampling procedures and some were leaders in the FSW community. It was ensured that these participants had not participated in the FGDs. In addition to the questions explored in the FGD, the semi-structured interviews also addressed possible delivery mechanisms for PrEP rollout and scale-up in Mysore and Mandya.

### Qualitative Data Collection and Analysis

The FGD and in-depth interview guides were translated from English to Kannada and interviews were conducted in Kannada, the local language. Interviews were audio-recorded. The Kannada recordings were transcribed directly into an English translation. Transcripts were coded for key themes and emergent categories, followed by thematic and content analysis [35] by a single coder who is familiar with the project. The key findings from the qualitative research were utilized to inform the phrasing of the quantitative questionnaire to ensure that questions were worded in a way that resonated with participants. Qualitative findings were also used to ensure that survey response options were exhaustive.

### Cross-Sectional Survey

A questionnaire was interviewer-administered to FSWs in Mysore and Mandya. The questionnaire was developed in English, translated into Kannada, and back-translated into English to ensure completeness. Respondent driven sampling (RDS) was employed to facilitate inclusion of and to capture the perspectives of more ‘hidden’ sex workers. The initial seeds were selected from diverse social networks to ensure representativeness in the sample. Eligible candidates were introduced to the study and interviewing procedures, after which informed consent was obtained and the questionnaire administered. The interviews were undertaken by the community researchers who underwent intensive training for two weeks under the supervision of a senior public health specialist. Seeds were asked to recruit their peers who met the study inclusion criteria. Referrals were screened for inclusion/exclusion criteria by the study coordinator and supervisors were responsible for ensuring that the entire protocol (from issuing coupon to administration of consent and interview) was adhered to. The supervisors and the study coordinator were also responsible for quality checks of the data. An initial eight seeds were given five coupons to distribute to five potential participants. Based on previous studies, it was understood that three waves are optimal for the sex work context in Mysore and Mandya. After the initial seeds, participants from waves 1 and 2 were given three coupons each to distribute to potential participants. To avoid duplication of intake, all participants were given a unique participant ID, and this information was recorded and tracked for duplication. The questionnaire was designed to collect information on demographic characteristics; knowledge, interest, perceptions, beliefs, and concerns around PrEP; acceptability and willingness for PrEP uptake; HIV-related risk and vulnerability (e.g. HIV knowledge, misconceptions, behaviour and HIV testing practices); barriers to PrEP uptake and adherence; facilitators to PrEP uptake and adherence; inclusion and exclusion criteria for enrollment into the demonstration project; and delivery mechanisms for PrEP roll-out and scale up.

### Statistical Analysis

Both continuous and categorical data were analysed using SPSS (IBM, Version 22). Univariate descriptive analysis was conducted to summarise the data and present frequency distributions and means.

### Ethical Considerations

Following approval from the World Health Organization, the research protocol was approved by the University of Manitoba’s Institutional Ethics Review Board (IERB) and the Institutional Ethics Committee (IEC) at *Durbar Mahila Samanwaya Committee* (DMSC), Kolkata, a sex worker organization that operates the *Sonagachi* project. All participants provided their written consent to participate in the study and the consent process was approved by both Research Boards.

## Results

### Participant Socio-Demographic Characteristics

From January to April 2015, 35 in-depth interviews were held with FSWs and 6 focus group discussions took place. The average age of FSW participants in the in-depth interviews was 34 years (age range = 25–45 years) and they had been practicing sex work for an average of 11 years (range = 1–20 years). Thirty-eight women participated in the six focus group discussions. The average age of FGD participants was 35 years (age range = 23–47 years) and they had been practicing sex work for an average of 9 years (range = 2–25 years).

A total of 427 FSWs (including 8 seeds) participated in the cross-sectional survey. Eight seeds were used to recruit 419 participants. The mean age of survey participants was 33 years (range = 19–49 years). The majority (95%) of the FSWs reported using multiple means for solicitation of clients (e.g., cell phone, public places, homes, through brokers). More than 90% of the FSWs reported entertaining clients in more than one venue (e.g., rented home, client’s home, motel, lodge, vehicle). The diversity in solicitation practices, as well as practices associated with the provision of sexual services makes it difficult to characterise sex-work related typology into distinct categories. Socio-demographic and sex work related characteristics of survey participants are presented in Tables 1 and 2.

**Table 1:** Socio-demographic characteristics of surveyed FSWs (N = 427).

Characteristic	n	%
**Age (years)**		
Mean(SD)	32.72(5.5)	
< = 25	56	13.1
26–35	226	52.9
36–45	143	33.5
>45	2	0.5
**Place of Residence**		
Rural	193	45.2
Urban	234	54.8
**Able to read and write**		
Yes	256	60.0
No	171	40.1
**Highest level of education**		
No formal schooling	174	40.8
Primary school (1–4)	58	13.6
Higher primary school (5–7)	83	19.4
High school (8–10)	88	20.6
PUC (11–12)	19	4.5
Higher education Undergraduate degree completed	4	0.9
Technical/Vocational School	1	0.2
**Current marital status**		
Divorced/separated/Unmarried—Living alone	19	4.5
Divorced/separated/Unmarried/Married—Living with husband	170	39.8
Married (live with partner other than husband)	99	23.2
Married—living alone	72	16.9
Widowed—live alone	45	10.5
Widowed—live with partner	22	5.2
**Age at first sex**		
Mean (SD)	17.12(2.9)	
<18	228	53.4
18–20	166	38.9
>20	31	7.3
Don’t know/Can't remember	2	0.5
**Age at first sex for money or gift**		
Mean (SD)	23.41(4.34)	
<18	17	4.0
18–25	291	68.2
>25	119	27.9
**Duration of practicing sex work-years**		
Mean (SD)	9.31(5.3)	
1–5	118	27.6
6–10	162	37.9
>10	147	34.4
**Place of solicitation**		
Rented home or friends' home	187	43.8
Through cell phone	330	77.3
Own home	36	8.4
Through a broker/pimp	153	35.8
Public places	195	45.7
Lodge, hotel, motel or bar	73	17.1
Dhaba (roadside restaurant)	26	6.1
Highway	115	26.9
Other	23	5.4
**Place where clients are entertained**		
Own home	85	19.9
Rented home or friend's home	388	90.9
Client's home	216	50.6
Public places	26	6.1
Lodge, hotel or motel	222	52.0
Dhaba	32	7.5
Client's vehicle	123	28.8
Other	37	8.67
**Number of clients entertained in a day**		
Mean(SD)	3(1.3)	
2–5 clients	400	93.7
>5	27	6.3
**Experience of a condom breaking in the past month**		
Yes	79	18.5
No	344	80.6

**Table 2:** Sex work and income of surveyed FSWs (N = 427).

**Amount of money rupees received per sex act in past one month**		
Mean (SD)	509.79(212.07)	
<500 (<$7.50 USD)	156	36.5
500 (<$7.50 USD)	161	37.7
>500 (>$7.50 USD)	110	25.8
**Amount of money (in Rs) received for overnight stay**		
Mean (SD)	1596.62(984.79)	
< = 1000 (< = $15 USD)	137	32.1
1001–2000 ($15–30 USD)	179	41.9
>2000 (>$30 USD)	39	9.1
No overnight stay	72	16.9
**Average monthly income in the past month (in Rs)**		
Mean (SD)	13309.86(5521.42)	
500–5000 ($7.50–75 USD)	31	7.3
6000–10000 ($90–149.50 USD)	131	30.7
11000–15000 ($164.50–224 USD)	158	37.0
>15000 (>$224 USD)	106	24.8
Refused	1	0.2

The findings (from the quantitative survey, in-depth interviews and FGDs) were broken down into the following main themes: (1) community interest and the need for PrEP; (2) prioritizing risk in participant selection for the PrEP demonstration project; and (3) the importance of experience and trust in addressing barriers to uptake and adherence. The findings for each theme are detailed below.

### Community Interest and the Need for PrEP

Following the PrEP awareness campaigns, participants demonstrated a good knowledge and understanding of PrEP.

*Some of them who are working in Ashodaya*. *They met me and told me about PrEP as a tablet and it will prevent HIV infection, but we also need to use condom…. She told me that PrEP is a tablet that can be given only to those who are negative and not HIV positive, but along with the tablet women should also use condom. She also said we will be provided training on it*. [*FSW, in-depth interview #8*]

*It is for my own safety. For myself. Something new is coming through the tablet and I feel excited about it. If there is something new that is good for our health, I feel I should take it*. [*FSW, in-depth interview #9*]

Participants expressed high levels of interest in PrEP and strongly associated its need to their experiences of risk. Almost all participants surveyed (95%, n = 406) expressed an interest in taking PrEP and among those, 89% stated that they would be willing to take PrEP every day. PrEP was seen as an exciting new tool in HIV prevention, one that would place control into the hands of the participants.

*We can prevent ourselves from getting infected by HIV. We see many people suffering because of HIV. So to take prior precaution we can take PrEP, which will prevent us from HIV. I was convinced about this medicine*. [*FSW, in-depth interview #4*]

PrEP was described as a prevention strategy that could potentially ease the stress and fear associated with instances where condom use was not possible, providing peace of mind to women in the knowledge that they are protected from contracting HIV.

*Yes. We want that tablet because it will prevent us from HIV. We used to be afraid that we will get HIV when some clients would have sex without condoms and sometimes when the condom gets torn. But if we take these tablets we need not worry about HIV. We can’t trust the clients to have safe sex. Our partners and boyfriends also refuse to wear condoms. This tablet will be useful for us to be fear free when we have sex with them even without condoms*. [*FSW, focus group discussion #5, Mysore*]

Furthermore, participants expressed that PrEP will allow them to maintain their health, thus protecting their earning potential and ability to support themselves, as well as their dependents. Many of the women interviewed had families for whom they were financially responsible. Participants viewed their earning potential as directly impacted by their health and they viewed taking PrEP as good for business.

*Yes. Most of the community wants to earn money, and they know they need to be healthy to do so. So, they want PrEP to stay healthy. They want it and after knowing how it can prevent HIV infections, they will also demand it*…. [*FSW, in-depth interview #2*]

…*I have the responsibility of earning and looking after my children and family. Many women are taking care of their children and aged people at home. Not all have a man providing for them. By taking these tablets I feel I will not get infected and so can earn and take care of my family. If I do not take it and I die of HIV then my children will face hardships*. [*FSW, in-depth interview #9*]

As PrEP in this setting is being recommended as a combination prevention strategy, alongside consistent condom use to prevent other STIs, it is important to gain an understanding of the perceived value of PrEP in the context of consistent condom use. Despite community norms of widespread condom use, there was still a feeling among community members that condoms alone are not enough.

*Now if I take this PrEP tablet, I will be safe even if someone forces me for sex [or] without a condom or even when the condom breaks. I will not have the fear because I know I have taken the precautionary tablet*… [*FSW, in-depth interview #1*]

Risk and vulnerability to HIV remains an inevitable experience shared by many sex workers. Forced sex and violence were reported to be far too common among the participants interviewed, making condom use impossible at times. One-quarter of the participants surveyed had experienced violence in the past twelve months and 8% reported experiencing forced sex during that time frame. Among the thirty-six participants surveyed who had experienced forced sex, condoms were known to be used only 42% of the time.

*Many times, apart from clients, lodge [motel] owners also have sex with us. They do not pay us, but have sex without condoms. Then they threaten that they will inform the police, or will not give a room to a client, etc. So I have sex without a condom so that they will continue to give rooms. Also if there is a raid and the police catch me, they take away all my money and also have free sex with me without a condom. So there are so many such occasions that I cannot avoid. Having PrEP tablets will help me greatly*. [*FSW, in-depth interview #14*]

When condoms are used, some clients intentionally remove or break condoms, placing women at risk of infection. Over one-third of surveyed participants reported experiencing condom breakage in the last year and 19% of all participants experienced breakage in the last month.

*I need it. Maybe once in a week while having sex, the condom may break and I may be exposed to risk of infection, so I need the tablet*. [*FSW, in-depth interview #14*]

Despite high rates of condom use with clients, sex without condoms with husbands, partners, and lovers is still common. Nearly all participants (97%) surveyed reported that they use a condom every time they have sex with occasional clients. Condom use with regular clients was slightly less consistent, with 85% of participants stating that they used a condom every time with regular clients. However, condom use with regular partners continues to be challenging. Of the 303 participants (71%) surveyed who reported having a regular non-paying sexual partner, only 29% expressed that they used a condom consistently, and 48% reported using a condom at the time of last sex. PrEP could play an important role in providing protection in these instances of unprotected sex.

*I have a lover from the past two years…. This person is now having sex with me without condoms from the past two years. He doesn’t know that I do sex work. I do it without his notice. He questions me if I insist that he use condoms. I may not have any health problems. But how can I trust him that he doesn’t have any problems? So, I will use condoms outside with other clients. But I have sex without condoms at home. So I will take the tablets that you are giving me so that I will be safe. It may so happen that I will get the infection from my lover*. *What will I do in such situations?* [*FSW, in-depth interview #6*]

PrEP and condoms used in combination was seen as providing a ‘double safety’ for participants. In the event that a condom breaks or a woman is pressured into sex without a condom, PrEP would provide an extra layer of protection against HIV transmission and some assurance.

*I don’t completely rely on condoms because due to its wear and tear there might be chances of STD or HIV infection. So for double safety I would prefer taking this tablet as well as use condoms*. [*FSW, in-depth interview #26*]

*To continue to be in good health. It is kind of double protection for me, using condoms as well as PrEP tablets. Sometimes while I am returning home from a client the auto driver also has sex with me. He doesn’t use a condom. So I need PrEP tablets to protect me from all such instances*. [*FSW, in-depth interview #14*]

Although participants expressed a need for PrEP, it is important to note that not all participants were open to using both PrEP and condoms. Some participants questioned the need for PrEP at all, as they have managed to effectively protect themselves from infections through other HIV prevention strategies.

… *PrEP prevents us only from HIV but condoms will prevent us from infecting STIs. If I don’t use condoms I will get different kinds of STIs. So we should use condoms even if we take PrEP. But I don’t think our community women will agree to use both*. [*FSW, in-depth interview #3*]

*As a community leader when I was educating our community about PrEP many asked me as to why they should take both PrEP and use condoms. Why can’t we use either of them? Many said they don’t take tablets even when they have fever but prefer some home remedies, they ask why should they take this tablet*. [*FSW, in-depth interview #29*]

*There may be few of them who would not agree to use them. They would ask if it is necessary to have it before having sex, why can’t we have sex without it, we were not using it till now*…. [*FSW, in-depth interview #1*]

Some participants expressed concern that PrEP use may undermine the community norm of condom use.

*I feel when we started talking about condoms, we stressed on its use saying we will not get HIV infection. Now if the tablets do the same, we will all feel that why use condoms when we are taking the tablets? Just like it is important to eat food regularly, we feel using condoms regularly is equally critical. But with the tablets, I feel people will lose interest in using condoms*. [*FSW, in-depth interview #9*]

In contrast, many survey participants reported that condom use would remain unchanged while taking PrEP (Table 3). Of participants surveyed, 99% stated that they would continue to use condoms while taking PrEP and 99% also claimed that they would use them as much or more than they do now.

**Table 3:** Condom use among surveyed FSWs who are willing to take PrEP.

Characteristic	n	%
**Continue to use condoms while taking PrEP (N = 424)**		
Yes	420	99.1
No	3	0.7
Refused	1	0.2
**Frequency of condom usage while taking PrEP (N = 420)**		
More than you do now	20	4.8
Same as you do now	396	94.3
Less than you do now	3	0.7
Refused	1	0.2
**Frequency of condom use with clients while taking PrEP (N = 420)**		
More condom use	18	4.3
Same condom use	389	92.1
Less condom use	14	3.3
Refused	1	0.2
**Frequency of condom use with regular partner while taking PrEP (N = 420)**		
More condom use	7	1.7
Same condom use	248	59.1
Less condom use	16	3.8
I don't have a regular partner	123	29.3
Refused	26	6.2

### Prioritizing Risk in Participant Selection

An important goal of the feasibility study was to explore the inclusion criteria for participants in the demonstration project. Although there was an overall feeling that all sex workers would benefit from PrEP, there was clear consensus that women who experienced more risk should be given priority for inclusion. Often, this meant younger sex workers, who attracted more clients, and also might be less knowledgeable about safer sex practices, less experienced with condom use, and less empowered to negotiate condom use.

*Like I have some friends who are [around] 25 years of age and they have a huge client load. They might use condoms with some of their clients and may not with some. As mentioned earlier they might have carried 4 or 5 condoms and unexpectedly they might go with much more clients where getting a condom would be impossible. In such cases these are the people under high risk and these are the people who should have PrEP tablets*. [*FSW, in-depth interview #30*]

One way suggested to judge “client load”, and thus risk, was based on how many condoms community members picked up. Younger and newer sex workers were also thought to be less experienced with proper condom use.

*Women who take more condoms means that they get more clients, irrespective of their age and so they must be given preference…. Young women [sometimes] do not know how to put a condom onto a man, and if they don’t do it correctly it may break… So they will be at high risk to HIV infections and so preference must be given to them. Women like me who take 3 clients a month maybe left out and given only if the tablets are available*. [*FSW, focus group discussion #1, Mysore*]

Among survey participants, 79% thought that the demonstration project should include FSWs under the age of 25, 58% prioritized the inclusion of FSWs who do not use condoms when clients offer more money, and 57% wanted to include FSWs who do not consistently use condoms with regular partners. Almost half (49%) of the survey participants wanted to include FSWs who drink alcohol regularly, and 48% prioritized those FSWs who do not use condoms consistently with clients. Almost one-third suggested including sex workers who have high client loads, 37% recommended including new FSWs who may not have the power to negotiate safer sex with clients, and 30% suggested the inclusion of those who travel frequently for sex work. According to the participants, all of these groups of FSWs are highly vulnerable and may compromise condom use, and therefore be at a higher risk for HIV.

Participants also stressed that attempts should be made to reach FSWs who work in home-based settings, who may be less likely to access services, may practice sex work in secret, and may be at higher risk for HIV infection. Home-based FSWs may be more vulnerable and can experience higher risk, as information and condoms often do not reach them. As their involvement in sex work is often hidden, they may be reluctant to come to the clinic to access services.

*Those doing this work at home or their brokers will not come forward to the office and mingling with our community will be comparatively less. As they basically prefer maintaining their work as secret. But those community [members] among the street will interact with other community [members] and information about this tablet will thereby pass on to one community to other. The ones who maintain secrecy about their work will hesitate to come forward and collect information on this tablet. For such people, PrEP is very useful and necessary*. [*FSW, in-depth interview #29*]

### The Importance of Experience and Trust in Addressing Barriers to Uptake and Adherence

Despite high levels of interest in PrEP, numerous potential barriers were cited for consideration prior to rolling out PrEP (see Table 4). Major concern was expressed around dosing; the need to take PrEP daily. The biggest obstacle facing PrEP may be the need for daily intake. One hundred and forty participants (33%) surveyed expressed daily intake as a challenge to PrEP use, and pointed to frequent travel and unpredictable work schedules as obstacles towards adherence. A desire for a weekly or monthly dosage, was often expressed.

*If you make it compulsory that we have to take it every day, it is difficult. We forget so many things that we have to do every day. Similarly we may forget taking the tablet also. Many times we are away for days together, we cannot carry the tablets with us wherever we go. Then we may miss it. That’s why I say if there are tablets that we can take once a week, then it is fine*…. [*FSW, in-depth interview #9*]

**Table 4:** Challenges in taking PrEP every day among surveyed FSWs (N = 424).

Challenges in taking PrEP every day	n	%
I might experience side effects	262	61.8
I might have difficulty taking PrEP as I consume alcohol	96	22.6
Others might see me take PrEP/ask questions about PrEP	200	47.2
My boyfriend/partner/lover might not give me permission to take PrEP	167	39.4
It is difficult to take tablets everyday	140	33.0
I live far, it will be difficult to travel to the office to collect tablets/for regular check-ups	110	25.9
I cannot afford to pay for my travel to the office	127	30.0
It will be difficult to take tablets when I am away on contract work	42	9.9
People might suspect that I have HIV	159	37.5
I might have difficulty in paying for PrEP medicine	43	10.1

The need for long-term adherence was also of concern. Participants pointed to challenges with medication compliance as an example of the difficulties that people have in finishing their course of treatment, once symptoms subside. These difficulties with long-term compliance are also to be expected when medication is prescribed for prevention purposes, such as is the case with PrEP.

*For example, I was suffering from severe fever I went to hospital and the doctor gave me some antibiotics and told me to take it for 5 days, I got those tablets but by then there was no fever or body pain. So I thought I will not take it as I was relieved from fever and rather decided to store it for the fever next time. Even though I know antibiotics have to be taken as this has been given for fever but still when I was relieved from fever, I thought it is not necessary to take antibiotics as they create drowsiness. With such attitude I think I might miss out the tablets*. [*FSW, in-depth interview #29*]

Notably, the trusted legacy of *Ashodaya*’s past work in health programs, such as condom promotion, screening and treatment for STIs, and reproductive health services, was often referenced as a key way of tackling these challenges.

*If we take time to talk to the community and make them understand that this is for their health and to keep them from getting HIV, then they will listen and take the tablets. In the beginning we did not use condoms, then, when Ashodaya came they taught about STIs, condoms, and encouraged us to go to ICTC [Integrated Counselling and Testing Centre] for HIV testing. Community understood it and followed. Similar methods must be used to convince them to take PrEP tablets*. [*FSW, in-depth interview #22*]

Privacy also becomes a major concern with daily intake, as participants expressed concern over challenges they might face in trying to hide PrEP use over an indefinite period of time from family and partners. Two hundred (47%) of participants surveyed named people seeing/questioning their PrEP use as a potential challenge. Participants anticipated facing questions from partners, family members, brokers, and clients. Some expected that partners would not allow women to take PrEP (39%). Some participants were concerned that others may suspect that they had HIV (38%), placing them at risk of violence, abandonment, and loss of income.

*Some clients may be close to us and open our purse. They may see the tablets within and ask us what they are and why we are taking it. They may suspect we are infected and so are on medication. People at home also open our purse and if they see tablets they also may get suspicious. Once when I had some tablets for my gas problem, client saw it and ran away thinking I have some disease. So carrying them with us is also a problem*. [*FSW, in-depth interview #9*]

Many women recommended strategies for addressing questions about daily intake based on their involvement in other health interventions. For instance, participants emphasized the need to extend information sessions to broader networks, including brokers and boyfriends, a process that had been undertaken in past education campaigns.

*There are possibilities that the brokers will not get clients for those women if they come to know that they are taking tablets. So we should first counsel the brokers and madams about the benefit of these tablets, so that they will know why women are taking the tablets*. [*FSW, focus group discussion #4, Mandya*]

*Participant: There may be problems especially at home because we have our family members around who would observe us. Same will be the case with boyfriends*.

Interviewer: How do we address that?

*Participant: We should conduct a meeting like we did initially about using condoms. We have to sensitise them and educate them about the tablets. We should conduct stakeholders meetings so that they will not create problems to women*. [*FSW, focus group discussion #5, Mysore*]

To address hesitations about new medications, it was suggested by participants to start the demonstration project by enrolling respected community leaders in order to build interest and trust in PrEP.

*We [community leaders] are the ones who are engaged in having sex with multiple partners. So we should be more conscious about our health and if we take these tablets we can ensure good health. First, we have to start using [PrEP] and once we know that it helps us, then we can spread that message to other community members who would in turn tell other women also. In this way all the community members will agree to take this tablet*. [*FSW, in-depth interview #4*]

*See what happens is, women are smart. If I tell them to start the tablets, they will say you take them first for three months and get tested. Show us the reports after three months and if we are convinced no infection is there, then we will also start on the tablets. So we [community leaders] need to demonstrate and show them that the tablets work*. [*FSW, in-depth interview #5*]

Once women are selected for the demonstration project, it will be important that special consideration is paid to pairing participants with outreach workers/community mobilizers (based on their individual preference) who will play a key role in promoting adherence. Pairings will be important, as most participants named specific individuals from whom they wanted to access PrEP. Surveyed participants interested in taking PrEP preferred to collect it from peer educators/community leaders (69%), the *Ashodaya* Clinic/Drop-In Centre (13%), delivered to their home (9%), or collected from a friend (8%). Confidentiality, privacy, and trust were the most important factors in choosing this person.

*We would like to take it from the women we know well like outreach workers, whom we meet regularly. We will like to take it from those whom we believe and trust. We can take from those women who work in Ashodaya. We trust all of them*. [*FSW, focus group discussion #4, Mandya*]

Other participants expressed a preference in accessing PrEP directly from *Ashodaya*’s doctor, as it was thought that the doctor would be able to respond to medical questions and side effects about treatment.

*I would like to take it from the doctor because he will examine me properly and then give the tablet. I will come to Ashodaya, meet the doctor and take it. I trust the doctor in Ashodaya. If I go elsewhere they may ask me pointed questions on what is wrong and what I did and so on. If I tell them I am a sex worker, I will be discriminated against. Here, the doctor knows I am a sex worker and I can tell my problems like what discharge or stomach ache etc., and I will be treated well*… [*FSW, in-depth interview #18*]

Support for those traveling far distances to access PrEP will need to be taken into consideration. PrEP availability should be individualized based on each participant’s preferences. Some women prefer to take a month’s supply, as they are not able to regularly come to the office, either because they live far away or because frequent visits may cause suspicion at home. Other women prefer to come daily or weekly, due to concerns that someone may find their tablets in the house and question them. Nearly half of participants (47%) surveyed wished to collect PrEP weekly, while about one-quarter preferred either daily or monthly pick-up.

*Since I am in the field everyday, I can get the tablets once a week or once in a month. I can keep the tablets at home in my cupboard which has a lock. I don’t have any problems in keeping a stock at home*. [*FSW, in-depth interview #3*]

*I live close by and so I can frequently take it. I cannot take it in bulk and stock at home. So I can take small dosages and keep coming back for more.… In some houses the husbands do not know what work we are doing. So if they come across the tablets they may get suspicious and find out about the tablet and questions will be asked why we are taking it. We cannot keep it in the house of relatives either. So we can keep it in our close friend’s house. If not, then I have to come every day to the office and take it. I am willing to do so*. [*FSW, in-depth interview #9*]

*I don’t have any problem in coming to the clinic. But in community members there are few of them who come from faraway places… It becomes difficult to follow up such people on their clinic visits and check-ups. Women who live in Mysore or nearby places would come to Mysore city for soliciting. It will not be a problem to those women but it will be tough for those who come from other places. We should think about them*… [*FSW, in-depth interview #3*]

As women are already working with outreach workers to access condoms and regular health screenings, PrEP pick-up can be negotiated to best meet the needs of individual participants based on existing routines.

Finally, participants raised questions about what will happen following the 16-month demonstration project period. Some have concerns about health consequences related to stopping PrEP after the trial. If PrEP were to remain free of cost (as it will be throughout the project), 86% of surveyed participants would be willing to take PrEP as long as required. Although 80% of surveyed women indicated that they would be willing to pay for PrEP out of pocket after the demonstration project, it was also clear that adherence might falter if women were expected to assume the cost. Table 5 shows the amount FSWs are willing to pay for PrEP after the demonstration project ends. Transition planning following the 16-month project period will be crucial if continued PrEP use is desired among the community.

**Table 5:** Willingness to pay for PrEP post demonstration project among surveyed FSWs (N = 424).

Willingness to pay in Indian rupees per day	n	%
Not willing to pay (0)	79	18.6
1–25 ($0.01–0.37 USD)	219	51.7
26–50 ($0.38–0.75 USD)	63	14.9
51–75 ($0.76–1.12 USD)	3	0.7
76–100 ($1.13–1.49 USD)	36	8.5
> 100 ($1.50)	20	4.7
Refused	4	0.9
Total	424	100.0

## Discussion

Some early PrEP studies among FSWs in parts of Asia and Africa were cancelled before they started due to a lack of community consultation and concerns about study protocols [5,31]. The feasibility study discussed in this manuscript was an important step in assessing interest and acceptance of PrEP before moving forward with a large-scale demonstration project in Mysore and Mandya districts, in Karnataka state, southern India. The study itself served to educate the community about PrEP. It was an opportunity to obtain community consent for the upcoming project, and as a means of gathering community insights regarding participant inclusion criteria and PrEP delivery mechanisms.

Participants in our study were drawn from members of a well-established community organization, and their familiarity and involvement with the organization shaped their understanding of risk and prevention programs, as well as their interest in participating in the demonstration project. Participants expressed their responses related to participant selection in terms of public health discourse surrounding risk, prioritizing women who may experience more HIV risk in their occupational environments for enrolment into the study, such as younger FSWs, those who do not use condoms regularly, and those who drink alcohol. These findings align with previous research that uncovered how collectivization surrounding interventions (structural, bio-medical, behavioural) shape community identities and collective security strategies [36]. Furthermore, qualitative research on PrEP use in Sub-Sahara Africa found that women placed a high level of importance on contributing to the well-being of their community [37]. Our findings point to the importance of situating PrEP scale-up within the trusted spaces of community-based organizations as a means of supporting PrEP uptake and adherence.

High interest in PrEP was linked to ongoing risks encountered by some sex workers that often create barriers to consistent condom use. PrEP offers an HIV prevention strategy that places control into the hands of its users, specifically sex workers themselves in our context. These findings are similar to other studies that have found that PrEP use increases a feeling of safety and empowerment during sex [38]. The data from the feasibility study shows the readiness, willingness, and strong desire for a PrEP demonstration project. Despite high levels of interest in PrEP, concerns were expressed regarding the potential stigma associated with being recognized as someone who takes pills every day; privacy around the administration of PrEP; the possibility of being identified as being HIV positive; potential adverse effects of a new medication; and challenges with daily drug adherence. These barriers mirrored those discussed in other acceptability studies with at-risk populations in Kenya [39, 40]. Within a clinical trial setting, counselling was identified as an important component to supporting adherence [39]. It was noted, however, that adherence was likely to suffer without the extra support systems present within a study setting. In a separate acceptability study, also in Kenya, community consultation among at-risk groups identified the importance of staff trained to address specific population needs as an adherence facilitator [40]. The findings from these studies point to the need for targeted PrEP interventions that are tailored to best meet community needs. In our study setting, *Ashodaya*’s extensive experience in rolling out community-based health interventions [27, 28, 41] will enable tailor-made approaches to PrEP delivery and adherence support, based on each individual participant’s needs and preferences. These adherence supports are especially important in the context of well-documented challenges to long-term medication adherence [40, 42]. Wider community consultations with other networks that govern sex work, such as boyfriends and brokers, will also be undertaken to address concerns over the stigma and discrimination that women may face while on PrEP.

There are several potential limitations to our study. As survey questions relied on self-reporting, it is possible that findings are subject to social desirability bias [43]. Furthermore, as focus group discussions, in-depth interviews, and surveys were conducted by community leaders, it is also possible that participants were more likely to feel pressure to speak favourably about PrEP. However, research has shown that the involvement of community researchers can facilitate the disclosure of sensitive information and improve the quality of data [44,45]. As participants expressed both interest and concerns about PrEP, it is likely that they felt comfortable disclosing their views during interview. Finally, as this research occurred within the context of a well-established sex worker collective, the findings of this study may not be generalizable to other settings where sex work occurs.

### Conclusion

As this paper helps to illustrate, community-organizations working with sex workers may be best-positioned to implement demonstration projects among FSWs due to their capacity, connections, and trust to reach those members who stand to benefit the most from access to PrEP. In our unique study setting, *Ashodaya* was able to draw on its past experience in HIV prevention and treatment programs to offer effective strategies to address community concerns surrounding uptake and adherence, including reaching out to broader social networks. It is imperative that if PrEP is to be introduced to female sex worker communities, it should only be done after adequate capacity building, awareness building, and demand creation. Situating PrEP scale up within the trusted spaces of community-based organizations should be considered as a means of supporting PrEP roll-out.

## Supporting Information

S1 File PrEP Feasibility Study Questionnaire. (XLSX)

## References

[1] UNAIDS. The Gap Report. 2014. Available from: http://files.unaids.org/en/media/unaids/contentassets/documents/unaidspublication/2014/UNAIDS_Gap_report_en.pdf.

[2] National AIDS Control Organization. National Integrated Biological and Behavioural Surveillance (IBBS) 2014–2015: High Risk Groups. December 2015. New Delhi: Ministry of Health and Family Welfare, Government of India. Available from: http://naco.gov.in/upload/2016%20Data/SIMU/IBBS%20Report%202014-15.pdf

[3] Cáceres CF, O’Reilly KR, Mayer KH, Baggaley R. PrEP implementation: moving from trials to policy and practice. *PrEP Implementation Science*: *State-of-the-Art and Research Agenda* 2015;1.

[4] Reza-Paul S, Beattie T, Syed HU, Venukumar KT, Venugopal MS, Fathima MP, et al Declines in risk behaviour and sexually transmitted infection prevalence following a community-led HIV preventive intervention among female sex workers in Mysore, India. *AIDS* 2008;22:S91–S100. 10.1097/01.aids.0000343767.08197.18 19098483

[5] Cáceres CF, Koechlin F, Goicochea P, Sow PS, O’Reilly K, Mayer K, et al The promises and challenges of pre-exposure prophylaxis as part of the emerging paradigm of combination HIV prevention. *Journal of the International AIDS Society* 2015;18(4 Suppl 3): 19949 10.7448/IAS.18.4.19949 26198341 PMC4509895

[6] Auerbach JD, Parkhurst JO, Caceres CF. Addressing social drivers of HIV/ AIDS for the long-term response: conceptual and methodological considerations. *Glob Public Health* 2011;6(Suppl 3):S293309. 10.1080/17441692.2011.594451 21745027

[7] World Health Organization. Guidance on pre-exposure oral prophylaxis (PrEP) for serodiscordant couples, men and transgender women who have sex with men at high risk of HIV: Recommendations for use in the context of demonstration projects. July 2012. 23586123

[8] World Health Organization. Pre-exposure prophylaxis. 2015. Available from: http://www.who.int/hiv/topics/prep/en/.

[9] Grant R, Lama JR, Anderson PL, MacMahon V, Liu AY, Vargas L, et al Preexposure chemoprophylaxis for HIV prevention in men who have sex with men. *New England Journal of Medicine* 2010;363(27):2587–2599. 10.1056/NEJMoa1011205 21091279 PMC3079639

[10] Thigpen MC, Kebaaberswe PM, Paxton LA, Smith DK, Rose CE, Segolodi TM, et al Antiretroviral preexposure prophylaxis for heterosexual HIV transmission in Botswana. *New England Journal of Medicine* 2012;367(5):423–34. 10.1056/NEJMoa1110711 22784038

[11] Baeten JM, Donnell D, Ndase P, Mugo NR, Campbell JD, Wangisi J, et al Antiretroviral prophylaxis for HIV prevention in heterosexual men and women. *New England Journal of Medicine* 2012;367(5):399–410. 10.1056/NEJMoa1108524 22784037 PMC3770474

[12] Choopanya K, Martin M, Suntharasami P, Sangkum U, Mock PA, Leethochawalit M, et al Antiretroviral prophylaxis for HIV infection in injecting drug users in Bangkok, Thailand (the Bangkok Tenofovir Study): a randomised, double-blind, placebo-controlled phase 3 trial. *The Lancet* 2013;381(9883):2083–90. 10.1016/S0140-6736(13)61127-7 23769234

[13] Van Damme L, Cornell A, Ahmed K, Agot K, Lombard J, Kapiga S, et al Preexposure prophylaxis for HIV infection among African women. *New England Journal of Medicine* 2012;367(5):411–22. 10.1056/NEJMoa1202614 22784040 PMC3687217

[14] Marrazzo J, Ramjee G, Nair G, Palanee T, Mkhize B, Nakabilito C, et al. Pre-exposure prophylaxis for HIV in women: daily oral tenofovir, oral tenofovir/emtricitabine or vaginal tenofovir gel in the VOICE study (MTN 003). 20th Conference on Retroviruses and Opportunistic Infections. Atlanta, GA, March 3–6, 2013.

[15] Karim QA, Karim SSA, Frolich JA, Grobler AC, Baxter C, Mansoor LE, et al Effectiveness and safety of tenofovir gel, an antiretroviral microbicide, for the prevention of HIV infection in women. *Science* 2010;329:1168–1174. 10.1126/science.1193748 20643915 PMC3001187

[16] Ndase P, Celum C, Campbell J, Bukusi E, Kiarie J, Katabira E, et al Successful discontinuation of the placebo arm and provision of an effective HIV prevention product after a positive interim efficacy result: the partners PrEP study experience. *Journal of Acquired Immune Deficiency Syndromes* 2014;66(2):206–212. 24828268 10.1097/QAI.0000000000000141

[17] McCormack S, Dunn DT, Desai M, Dolling DI, Gafos M, Gilson R, et al Pre-exposure prophylaxis to prevent the acquisition of HIV-1 infection (PROUD): effectiveness results from the pilot phase of a pragmatic open-label randomised trial. *The Lancet* 2015 10.1016/S0140-6736(15)00056-2. PMC4700047 26364263

[18] Haberer JE, Bangsberg DR, Baeten JM, Curran K, Koechlin F, Amico KR, et al Defining success with HIV pre-exposure prophylaxis: a prevention-effective adherence paradigm. *AIDS* 2015;500:14–01195. 10.1097/QAD.0000000000000647 PMC4480436 26103095

[19] Martin M, Vanichseni S, Suntharasamai P, Sangkum U, Mock PA, Leethochawalit M, et al The impact of adherence to preexposure prophylaxis on the risk of HIV infection among people who inject drugs. *AIDS* 2015;29(7):819–824. 10.1097/QAD.0000000000000613 25985403

[20] Medical Research Council Press Office. PROUD study interim analysis finds pre-exposure prophylaxis (PrEP) is highly protective against HIV for gay men and other men who have sex with men in the UK [press release]. London, UK 2014 Available from: http://www.ctu.mrc.ac.uk/news/2014/proud_statement_16102014.

[21] Cohen S, Vittinghoff E, Anderson P, Doblecki-Lewis S, Bacon O, Chege W, et al. Implementation of PrEP in STD clinics: high uptake and drug detection among MSM in the demonstration project. Conference on Retroviruses and Opportunistic Infections, Boston, MA: March 2014.

[22] Baeten J. Near elimination of HIV transmission in a demonstration project of PrEP and ART. Conference on Retroviruses and Opportunistic Infections, Seattle, WA: February 2015.

[23] Jana S, Basu I, Rotheram-Borus MJ, Newman PA. The Sonagachi Project: a sustainable community intervention program. *AIDS Education and Prevention* 2004;16(5):405–414. 10.1521/aeap.16.5.405.48734 15491952

[24] Basu I, Jana S, Rotheram-Borus MJ, Swendeman D, Lee SJ, Newman P, et al HIV prevention among sex workers in India. *Journal of Acquired Immune Deficiency Syndromes* 2004;36(3):845 15213569 10.1097/00126334-200407010-00012 PMC2826108

[25] Cornish F, Ghosh R. The necessary contradictions of ‘community-led’ health promotion: a case study of HIV prevention in an Indian red light district. *Social Science & Medicine* 2007;64(2):496–507. 17055635 10.1016/j.socscimed.2006.09.009

[26] Lorway R, Thompson LH, Lazarus L, du Plessis E, Pasha A, Fathima MP, et al Going beyond the clinic: confronting stigma and discrimination among men who have sex with men in Mysore through community-based participatory research. *Critical Public Health* 2013;24(1):37–87.

[27] Reza-Paul S, Lorway R, O’Brien N, Lazarus L, Jain J, Bhagya M, et al Sex worker-led structural interventions in India: a case study on addressing violence in HIV prevention. *The Indian Journal of Medical Research* 2012;135(1):98–106. 22382190 10.4103/0971-5916.93431 PMC3307193

[28] Lazarus L, Reza-Paul S, Pasha A, Jairan S, Hafeez Ur Rahman S, O’Neil J, et al Exploring the role of community-based peer support in improving access to care and antiretroviral treatment for sex workers in Mysore, India. *HIV & Social Services* 2012; 11:152–168.

[29] Argento E, Reza-Paul S, Lorway R, Jain J, Bhagya M, Fathima MO, et al Confronting structural violence in sex work: lessons from a community-led HIV prevention project in Mysore, India. *AIDS Care* 2011;23(1):69–74. 10.1080/09540121.2010.498868 21218278

[30] Dixon V, Reza-Paul S, D’Souza FM, O’Neill J, O’Brien N, Lorway R. et al Increasing access and ownership of clinical services at an HIV prevention project for sex workers in Mysore, India. *Global Public Health* 2012;7(7):779–791. 10.1080/17441692.2012.668918 22424476

[31] Syvertsen JL, Bazzi AMR, Scheibe A, Adebajo S, Strathdee SA, Wechsberg WM. The promise and peril of Pre-Exposure Prophylaxis (PrEP): using social science to inform PrEP interventions among female sex workers: review article. *African Journal of Reproductive Health*: *Special Edition on HIV/AIDS* 2014;3(18):74–83. PMC4605136 26050379

[32] Bowen DJ, Kreuter M, Spring B, Cofta-Woerpel L, Linnan L, Weiner D et al How we design feasibility studies. *American Journal of Preventive Medicine* 2009; 36(5):452–7. 10.1016/j.amepre.2009.02.002 19362699 PMC2859314

[33] Morse JM. Approaches to qualitative-quantitative methodological triangulation. *Nursing Research* 1991;40(2):120–3. 2003072

[34] Johnson RB, Onwuegbuzie AJ, Turner LA. Toward a definition of mixed methods research. *Journal of Mixed Methods Research* 2007;1(2):112–33.

[35] Boyatzis RE. Transforming qualitative information: Thematic analysis and code development. Sage; 1998.

[36] Lorway R, Khan S. Reassembling epidemiology: Mapping, monitoring and making-up people in the context of HIV prevention in India. *Social Science & Medicine* 2014;112:51–62. 24797356 10.1016/j.socscimed.2014.04.034

[37] Amico KR, Wallace M, Bekker LG, Roux S, Atujuna M, Sebastian E, et al Experiences with HPTN 067/ADAPT Study-Provided Open-Label PrEP Among Women in Cape Town: Facilitators and Barriers Within a Mutuality Framework. *AIDS and Behavior* 2016;17:1–5. 10.1007/s10461-016-1458-y PMC5378745 27317411

[38] Grant RM, Koester KA. What people want from sex and preexposure prophylaxis. *Current Opinion in HIV and AIDS* 2016;11(1):3–9. 10.1097/COH.0000000000000216 26569183 PMC6446930

[39] Van der Elst EM, Mbogua J, Operario D, Mutua G, Kuo C, Mugo P, et al High acceptability of HIV pre-exposure prophylaxis but challenges in adherence and use: qualitative insights from a phase I trial of intermittent and daily PrEP in at-risk populations in Kenya. *AIDS and Behavior* 2013;17(6):2162–72 10.1007/s10461-012-0317-8 23080358 PMC3690654

[40] Mack N, Odhiambo J, Wong CM, Agot K. Barriers and facilitators to pre-exposure prophylaxis (PrEP) eligibility screening and ongoing HIV testing among target populations in Bondo and Rarieda, Kenya: Results of a consultation with community stakeholders. *BMC Health Services Research* 2014;14(1):231. 24886646 10.1186/1472-6963-14-231 PMC4051373

[41] Chevrier C, Khan S, Reza-Paul S, Lorway R. ‘No one was there to care for us’: *Ashodaya* Samithi's community-led care and support for people living with HIV in Mysore, India. *Global Public Health* 2015:1–4. 10.1080/17441692.2015.1091488 26548553

[42] Amico K. Rivet and Stirratt Michael J. Adherence to Preexposure Prophylaxis: Current Emerging and Anticipated Bases of Evidence. *Clinical Infectious Diseases* 2014;59(S1):S55–60. 24926036 10.1093/cid/ciu266 PMC4060253

[43] Catania JA, Gibson DR, Chitwood DD, Coates TJ. Methodological problems in AIDS behavioral research: influences on measurement error and participation bias in studies of sexual behavior. *Psychological Bulletin* 1990;108(3):339 2270232 10.1037/0033-2909.108.3.339

[44] Coupland H, Maherb L, Enriquez J, Khanh L, et al Clients or colleagues? Reflections on the process of participatory action research with young injecting drug users. *International Journal of Drug Policy* 2005;16:191–98.

[45] Schnarch B. Ownership, control, access, and possession (OCAP) or self-determination applied to research: A critical analysis of contemporary First Nations research and some options for First Nations communities. *International Journal of Indigenous Health* 2004;1(1):80.

